# Should patient enrollment criteria for anti‐VEGF phase III trials be reconsidered

**DOI:** 10.1111/1753-0407.13468

**Published:** 2023-09-20

**Authors:** Joel Hanhart, Sohee Jeon, Raimo Tuuminen

**Affiliations:** ^1^ Department of Ophthalmology, Shaare Zedek Medical Center Jerusalem Israel; ^2^ Hebrew University of Jerusalem Jerusalem Israel; ^3^ Vitreoretinal Service Keye Eye Center Seoul South Korea; ^4^ Department of Ophthalmology Kymenlaakso Central Hospital Kotka Finland; ^5^ Faculty of Health Sciences Ben‐Gurion University of the Negev Beer‐Sheva Israel; ^6^ Helsinki Retina Research Group, Faculty of Medicine University of Helsinki Helsinki Finland

Dear Editor,

Bloomgarden reviewed a variety of relevant studies summarizing new approaches to the treatment of diabetes citing a study showing that anti‐vascular endothelial growth factor (VEGF) treatment of over 14 000 patients with diabetic retinopathy (DR) was associated with increased likelihood of myocardial infarction (MI) and overall cardiovascular disease (CVD), although with the annotation that the association may be confounded by the relationship between cardiovascular comorbidities and more severe degrees of DR.^1^

Indeed, in a recent retrospective longitudinal population‐based analysis of 1 731 782 individuals, Zafar et al reported that, in patients with DR, intravitreal anti‐VEGF injections were associated with an increase in the likelihood of systemic adverse events, such as acute MI, CVD, or kidney disease.^2^ This association was maintained when controlling for factors such as age, sex, race, ethnicity, smoking status, mean hemoglobin A_1c_ levels, statin use, DR severity, and comorbidity burden. The 5‐year cumulative incidence of any systemic adverse event was 37.0% (5187/14022) in the injection group versus 18.4% (316753/1717760) in the noninjection group (*p* < .001).^2^ Anti‐VEGF injections were independently associated with a higher likelihood of developing any systemic adverse event (odds ratio, 1.8; 95% confidence interval [CI], 1.7–1.9) when controlling for age, race, sex, ethnicity, tobacco use, severity of DR, Deyo‐Charlson Comorbidity Index score, mean hemoglobin A_1c_, total number of injections, and statin use. Some potential biases that may have been confounding factors associated with the results were mentioned in the Invited Commentary by VanderBeek.^3^ However, other studies in the clinical practice setting, sometimes termed “real‐world” studies, also indicate that intravitreal anti‐VEGF injections after acute MI or CVD may be associated with increased mortality.^4^, ^5^, ^6^ After the reports of increased mortality rates from several registration trials, patients who had experienced a cardiovascular or cerebrovascular event were excluded from studies for intravitreal anti‐VEGF injection trials for DR.^5^, ^7^, ^8^ In addition, for age‐related macular degeneration, registration trials with anti‐VEGF agents specifically excluded patients with DR.^9^, ^10^, ^11^, ^12^, ^13^

Unlike patients reported in registration and other randomized clinical trials, patients with DR in the clinical practice setting are possibly at greater risk of serious adverse effects.^14^, ^15^, ^16^, ^17^, ^18^, ^19^, ^20^ Those patients are presumably at risk of adverse effects that might remain undetectable should the design of registration trials not consider the results of large clinical practice setting studies. DR patients who reflect real‐world cohorts should, then, be included in phase III trials for better information on the risks of severe adverse effects of anti‐VEGF use.

## CONFLICT OF INTERESTS STATEMENT

Joel Hanhart and Sohee Jeon have nothing to disclose. Raimo Tuuminen is a scientific advisor (advisory board, honoraria) to Alcon Laboratories, Inc., Allergan, Inc., and has received clinical trial support (study medicines) from Bayer AG and Laboratoires Théa.
